# Incidence, prevalence, and treatment of anemia of non-dialysis-dependent chronic kidney disease: A retrospective database study in France

**DOI:** 10.1371/journal.pone.0287859

**Published:** 2023-07-05

**Authors:** Karim Dardim, Jérôme Fernandes, Arnaud Panes, Julien Beisel, Aurélie Schmidt, Josephine Wolfram, Lora Todorova, Laurence Dubel, Thierry Lobbedez

**Affiliations:** 1 Association Limousine pour l’Utilisation du Rein Artificiel à Domicile (ALURAD), Limoges, France; 2 Centre Hospitalier de la Côte Basque, Bayonne, France; 3 HEVA, Lyon, France; 4 Astellas Pharma Europe B.V., Leiden, The Netherlands; 5 Astellas Pharma France, Levallois-Perret, France; 6 University Hospital, Caen, France; Royal College of Surgeons in Ireland, IRELAND

## Abstract

**Background:**

Minimal data are available regarding the prevalence and incidence of anemia among patients with non-dialysis-dependent chronic kidney disease (NDD-CKD) in France.

**Methods:**

This was a retrospective non-interventional study of patients with a record of NDD-CKD in the Echantillon Généraliste des Bénéficiaires (EGB) database between January 01, 2012, and December 31, 2017. The primary objective was to estimate the annual incidence and prevalence of anemia of NDD-CKD. Secondary objectives included description of the demographics and clinical characteristics of patients with NDD-CKD-related anemia. An exploratory objective was to use machine learning to identify patients from the general population that might have NDD-CKD but without a recorded ICD-10 diagnosis of CKD.

**Results:**

During 2012–2017, 9865 adult patients in the EGB database had confirmed NDD-CKD; of these, 49.1% (4848/9865) had anemia. From 2015 to 2017, estimates of incidence (108.7–114.7 per 1000 population) and prevalence (435.7−449.5 per 1000 population) of NDD-CKD-related anemia were stable. Less than half of patients with anemia of NDD-CKD were treated with oral iron, and approximately 15% were treated with erythropoiesis-stimulating agents. Based on adult French population projections in 2020 and an estimated prevalence rate in 2017 of 42.2 per 1000 population for confirmed plus possible NDD-CKD (as a proportion of the general French population), the estimated number of patients with possible NDD-CKD in France was 2,256,274, approximately five-fold greater than the number identified by diagnostic codes and hospitalizations.

**Conclusions:**

Anemia of NDD-CKD was shown to be a constant long-term burden in France, and its apparent prevalence may still be significantly underestimated. Given the potential treatment gap, additional initiatives to better identify and treat NDD-CKD anemia may improve patient management and treatment outcomes.

## Introduction

Non-dialysis-dependent chronic kidney disease (NDD-CKD) is associated with a high burden of comorbidity, adverse clinical outcomes, and mortality [1–5]. A global meta-analysis of observational studies has estimated the global prevalence of NDD-CKD (stages 1–5) in the general adult population to be 13.4% (95% confidence interval [CI]: 11.7–15.1) [6].

Anemia is a common complication of CKD [7, 8] that develops in the early stages of the disease and increases with disease severity, affecting up to 90% of patients with stage 5 CKD [9, 10]. In a recent systematic literature review, anemia was consistently associated with greater mortality, hospitalizations, major adverse cardiovascular events (MACE), and CKD progression [9]. CKD patients with comorbid anemia and cardiovascular disease have been shown to have a significantly diminished quality of life [11].

The substantial burden of NDD-CKD has led to its recognition as a public health priority, with increased awareness, early detection, and active intervention being important considerations in helping to delay or prevent complications [1, 3, 12, 13]. The Global Burden of Disease (GBD) study estimated approximately 6 million cases of CKD in France in 2017, which resulted in over 9,000 deaths [3]. A further study conducted in France by Stengel et al. in 2011 reported a CKD prevalence among the elderly (≥65 years) of 27.9%, with estimates of 7.0% for stages 1–2, 16.7% for stage 3a and 4.2% for stage 3b or higher [14]. Additionally, in an international collaborative study from 2013 to 2018 in patients with NDD-CKD stages 3–5, 28% of patients in France were reported to have hemoglobin (Hb) levels <12 g/dL [15]. However, developing disease management strategies at the population level requires reliable estimates of prevalence. Globally, and in France in particular, there is a paucity of data exploring the prevalence of NDD-CKD and related anemia in the general population; consequently, the lack of reliable prevalence estimates complicates the development of corresponding management strategies at the population level. Prevalence of anemia has been shown to increase with CKD progression [10]; however, this has not been evaluated extensively in French NDD patients with anemia of CKD”.

To provide greater insight into NDD-CKD and associated anemia in France, this retrospective study investigated the epidemiology and treatment of anemia among NDD-CKD patients. This study used real-world data from Echantillon Généraliste des Bénéficiaires (EGB), a health insurance database to act as a representative sample of the healthcare consumption of the French population. The EGB is a well-established and extensive resource for epidemiology studies in France, particularly for those focused on the long-term research of non-rare diseases [16–20]. However, in the absence of a confirmed CKD diagnosis, there is an absence of methodology or data in the EGB database to identify CKD patients from their biological results and outpatient medical diagnoses are not captured. Consequently, a machine learning-derived algorithm was also applied to estimate the number of patients in France who may have undiagnosed NDD-CKD and related anemia.

## Materials and methods

### Ethics

EGB is a medico-administrative database of insured persons, and all patient-level data used for this retrospective analysis were collected as part of routine diagnosis and treatment. A unique anonymous identification number was associated to each insured person; as such, all data were fully anonymized prior to access and inclusion in this study. The Health Data Hub (a French public structure), and an independent scientific committee approved the access and use of the EGB to conduct this study on April 7, 2020 (dossier n°778539). Informed consent was not sought.

### Study design and patients

This was a non-interventional, retrospective database study in France using the EGB database, which constitutes a 1/97^th^ random sample from the Système National d’Information Interrégimes de l’Assurance Maladie/Système National des Données de Santé (SNIIRAM/SNDS) database [21], selected through the National Insurance Recording System. Access to the EGB does not require approval from the Commission Nationale de l’Informatique et des Libertes as there is a simplified process in place that requires approval from an independent scientific committee and the Health Data Hub [22].

The SNIIRAM/SNDS database includes all ambulatory care, hospital stay reimbursement data and death-related data nationwide in France [23]. Furthermore, the SNIIRAM/SNDS database links, at the patient level, data for all outpatient-reimbursed health expenditures and hospitalizations in all public and private hospitals. Currently, the SNIIRAM/SNDS database covers 98.8% of the French population and provides information on patient demographics, long-term diseases, universal health coverage, clinical events, procedures, medicine prescriptions, laboratory test dates, hospitalizations, and cost coding [21, 23]. The SNIIRAM/SNDS database does not include laboratory test results, coding for physician specialties, most inpatient drug records, or outpatient diagnoses.

**Fig 1:**
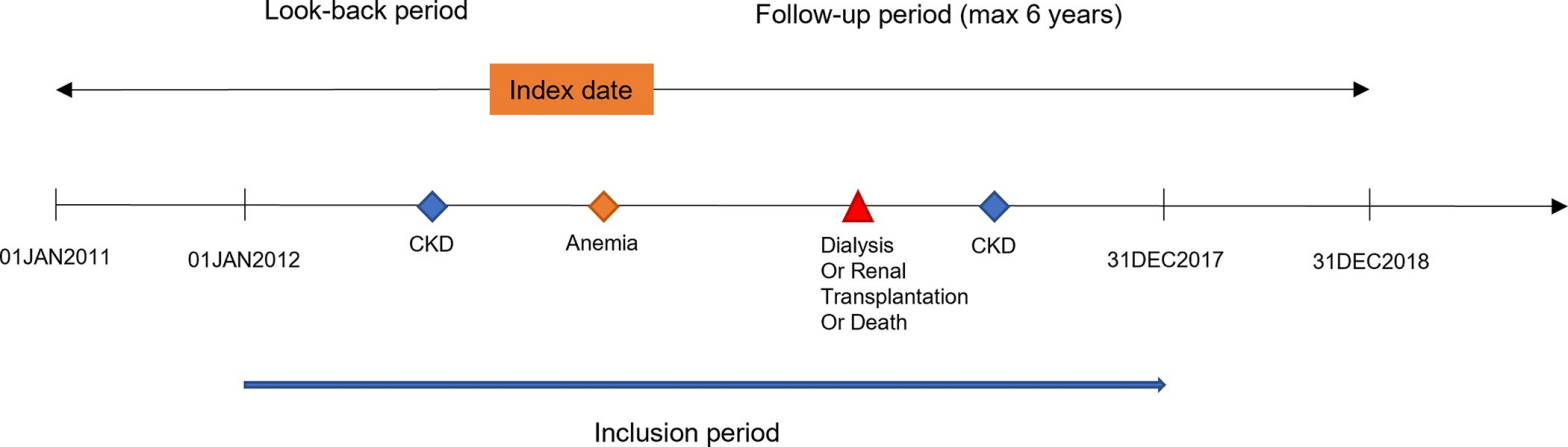
Study design.

**Fig 2:**
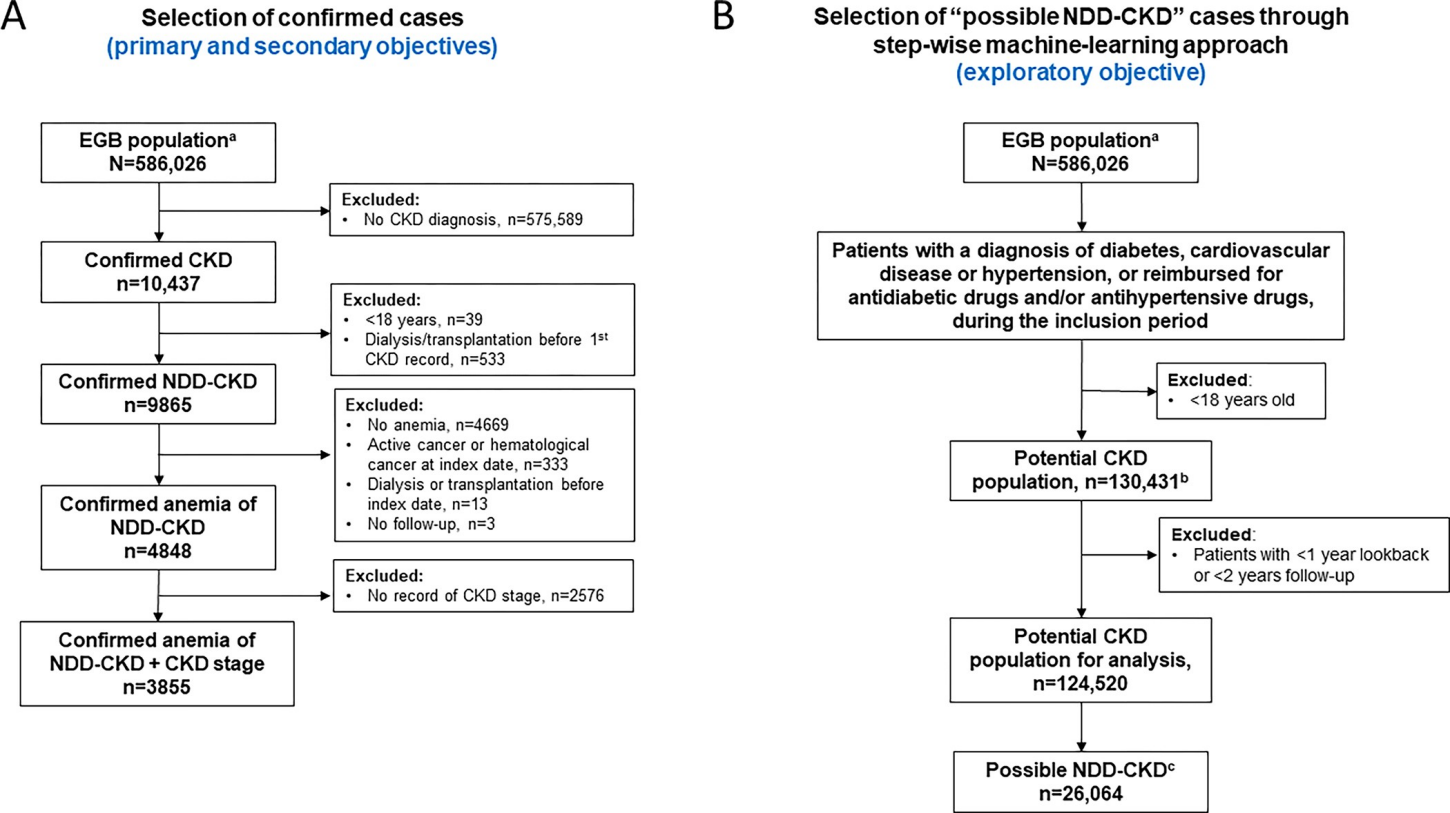
Study populations. A) Selection of confirmed cases (primary and secondary objectives); B) Selection of “possible NDD-CKD” cases through step-wise machine-learning approach (exploratory objective). ^a^Adult patients between 2012 and 2017. ^b^Patients with diabetes, cardiovascular disease, or hypertension between 2012 and 2017. ^c^Determined through use of machine-learning algorithm. CKD, chronic kidney disease; EGB, Echantillon Généraliste des Bénéficiaires; NDD, non-dialysis-dependent.

The study inclusion period was from January 01, 2012, to December 31, 2017. Eligible patients were adults aged ≥18 years with confirmed NDD-CKD, defined as ≥1 hospitalization with a CKD diagnostic code and/or those who were covered ≥1 day with a long-term disease registration (“affection de longue durée” [ALD]) for CKD diagnosis, during the inclusion period. Patients were excluded if they were aged ≤18 years at first CKD record or underwent dialysis or renal transplantation before the first CKD record (**Figs 1 and 2A**).

Anemia was identified as ≥1 reimbursement of drugs for anemia (iron [intravenous or oral] and/or erythropoiesis-stimulating agents [ESAs]) or ≥1 hospitalization with an anemia diagnosis during the inclusion period. The index date (ID) for patients with NDD-CKD-related anemia was defined as their first record of anemia during the inclusion period. No ID was defined for patients with NDD-CKD and unknown anemia status.

Patients with a record of chemotherapy or hematological cancer ≤1 year prior to their ID were excluded from the analysis. Patients were followed until dialysis, renal transplantation, death, end of affiliation of French health insurance, or end of study period, whichever occurred first. For the main statistical analysis, patients were followed retrospectively (lookback period) for the maximum time available between their ID and January 01, 2011 (**Fig 1**). Data from 2011 onwards were used to ensure a minimum lookback period of 1 year for each patient to assess whether their anemia was incident or prevalent. Further details to ensure that the EGB database would provide a sufficient sample size for our study are provided in the Supplementary Methods section in **S1 File**.

### Study objectives

The primary objective was to estimate the incidence and prevalence of anemia among patients with confirmed NDD-CKD. Secondary objectives were to (i) estimate the incidence and prevalence of NDD-CKD among the general population in France; (ii) estimate the incidence and prevalence of anemia of NDD-CKD among the general population in France; (iii) describe the demographics and clinical characteristics of incident and prevalent patients with anemia of NDD-CKD, including disease stage (defined according to ICD 10 codes, which align with KDIGO 2012 guidelines [24]), presence of cardiovascular disease, and diabetes; (iv) describe the therapeutic management of incident patients with anemia of NDD-CKD over the first year after the ID (oral iron and ESA treatment); and (v) describe the disease progression of incident patients with anemia of NDD-CKD during follow-up. Further details regarding the estimation of incidence and prevalence in these populations are included in the Supplementary Methods section in **S1 File**.

Cardiovascular disease included coronary artery disease, heart failure, arrhythmias, occlusive peripheral arterial disease of the lower limbs, abdominal aortic aneurysm, and history of stroke. Disease progression was defined as transplantation, dialysis, MACE+ (a composite endpoint of all-cause mortality, stroke and myocardial infarction plus heart failure requiring hospitalization and unstable angina requiring hospitalization), and/or death.

An exploratory objective was to use machine learning to identify patients from the general population that might have NDD-CKD (defined as “possible” NDD-CKD) but without a recorded ICD-10 diagnosis of CKD, and to estimate the number of patients with anemia in the combined “confirmed + possible” NDD-CKD population.

### Data extraction and statistical analyses

Data were summarized descriptively, and no significance testing was performed. Data analysis incorporated feedback from an expert scientific committee that included a pharmacist, a nephrologist, and a coding clinician. Demographic and clinical characteristics (sex, presence of a CKD long-term disease, CKD stage, diabetes, and cardiovascular disease status) were reported as categorical variables. Age was described as a continuous variable. For the primary analysis, an incident NDD patient with anemia of CKD was defined as a patient with a first marker of anemia during the follow-up period and without a prior marker of anemia since 2011. Once a patient was identified as anemic in a given year, the patient was considered anemic for the rest of the follow-up period. Sensitivity analyses were conducted to calculate incidence and prevalence rates using a 3-year lookback period prior to the calendar year of analysis. To estimate incidence and prevalence among the general population in France, the results of the French 2020 census were used. Epidemiological endpoint analyses were repeated for the pooled “confirmed + possible” NDD-CKD group identified by the machine learning tool.

Route of iron administration (intravenous/oral) and ESA type were described. The occurrence of and time to disease progression were estimated using Kaplan–Meier methods. Patients were censored after 5 years of follow-up. No missing data were identified, as the EGB database included all incidents of reimbursed healthcare. Data extracted in the EGB for the study were subject to quality control and consolidation prior to being made available. As such, in the variables accessible for the study, it was not expected to find missing values and further replacement or imputation was not carried out.

### Identification of patients with “possible” NDD-CKD using machine learning

To complement the confirmed population of NDD-CKD patients identified by diagnostic codes and hospitalizations, a machine learning algorithm was developed to identify patients with “possible” NDD-CKD but who could not be captured using the case definition of NDD-CKD. Details of the machine learning algorithm have been described previously [25]. Briefly, a “potential” NDD-CKD population was identified from the EGB database, comprising patients with a diagnosis of diabetes, cardiovascular disease, or hypertension or patients reimbursed for antihyperglycemic and/or hypertensive drugs during the inclusion period; only patients with ≥1 year of lookback or ≥2 years of follow-up were included for analysis (**Fig 2B**). A “possible” NDD-CKD population was then extracted from this “potential” NDD-CKD analysis population using an unsupervised machine learning algorithm (One Class Support Vector Machine, using scikit-learn Python 0.23.2 and Python 3.7), which was used to find NDD-CKD patients based on potential NDD-CKD-related variables: sex, number and duration of hospitalizations for renal diseases, number of general practitioner visits, medications, and number/type of biological examinations. A distance metric between patients was defined based on these variables, and was used to determine similarity between patients. The algorithm then learned to construct a non-linear boundary around the potential NDD-CKD population to create a decision rule for possible NDD-CKD versus non-NDD-CKD, with outliers considered possible NDD-CKD. The algorithm was refined with input from the expert scientific committee. Further details for the step-by step selection, training and validation process for the machine learning algorithm are provided in the Supplementary Methods section in **S1 File**.

## Results

### Patient selection

**Fig 3:**
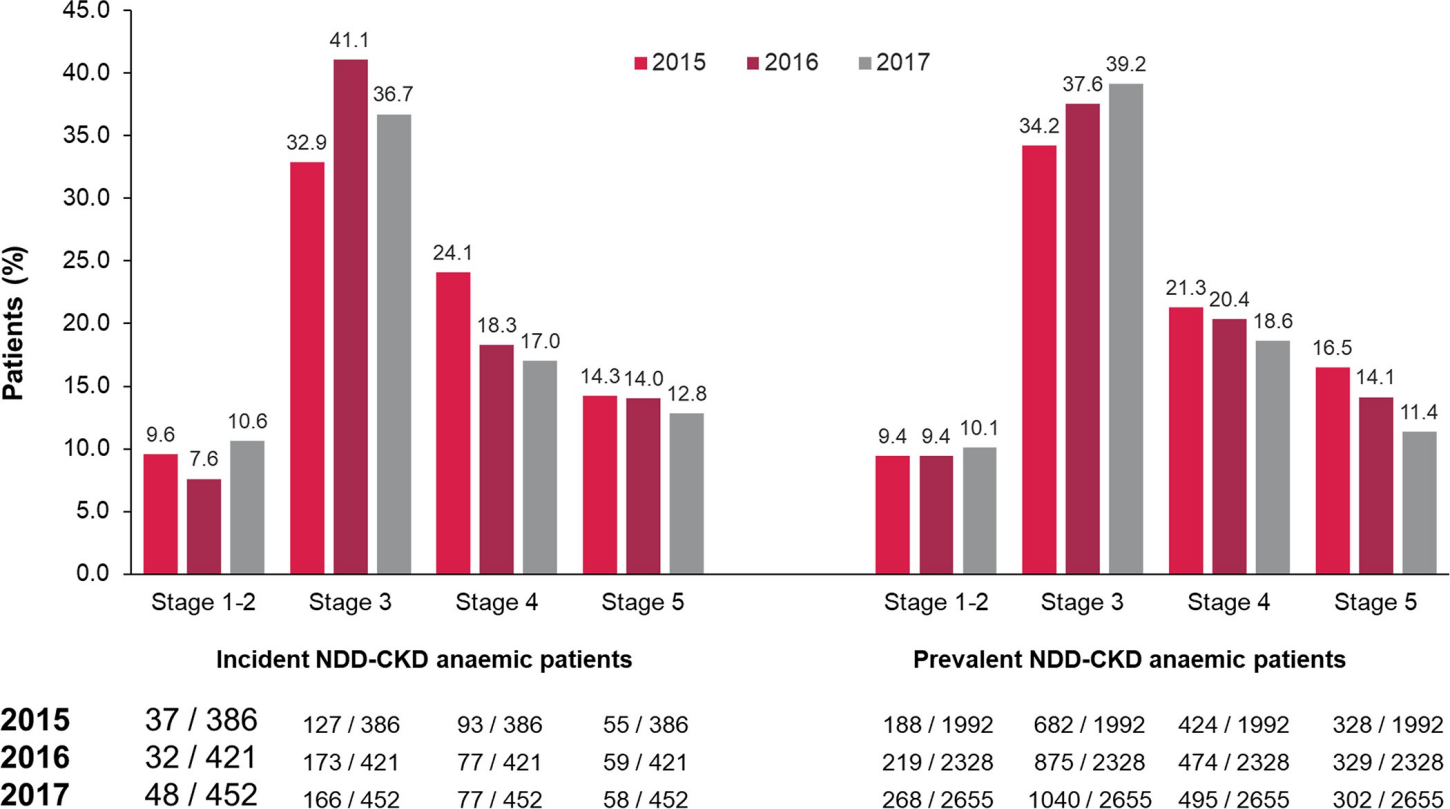
CKD stage of patients with incident and prevalent anemia of NDD-CKD in the EGB database. CKD stage not available for all patients. Incident missing n/total N (%): 2015, 74/386 (19.2%); 2016, 80/421 (19.0%), 2017, 103/452 (22.8%). Prevalent missing n/total N (%): 2015, 370/1,992 (18.6%), 2016, 431/2,328 (18.5%), 2017, 550,2,655 (20.7%). CKD, chronic kidney disease; EGB, Echantillon Généraliste des Bénéficiaires; NDD, non-dialysis-dependent.

Among patients extracted from the EGB database, 9865 adults had confirmed NDD-CKD from 2012 to 2017. Approximately half (n = 4848, 49.1%) of these patients had anemia and one-third (n = 3855, 39.1%) had anemia plus records confirming their CKD stage (**Figs 2A, 3**).

### Incidence and prevalence of anemia of NDD-CKD

From 2015 to 2017, the incidence and prevalence of anemia among patients with NDD-CKD in the EGB database were stable (incidence: 108.7−114.7 per 1000 population; prevalence: 435.7−449.5 per 1000 population) (**Table 1**, main analysis). Furthermore, 69.2% of prevalent NDD patients with anemia of CKD in the EGB population in 2017 had CKD stages 3–5 (**Fig 3**).

**Table 1:** Incidence and prevalence of anemia among patients with NDD-CKD in the EGB database per year (2015–2017).

Year	Number of incident NDD patients with anemia of CKD	Number of NDD-CKD patients at risk of anemia^a^	Incidence^b^, estimate per 1000 population (95% CI)	Number of prevalent NDD patients with anemia of CKD	Number of NDD-CKD patients at risk of anemia	Prevalence^c^, estimate per 1000 population (95% CI)
Main analysis^d^						
2015	386	3366	114.7 (104.3–125.9)	1992	4572	435.7 (421.4–450.1)
2016	421	3818	110.3 (100.7–120.6)	2328	5321	437.5 (424.2–450.9)
2017	452	4160	108.7 (99.6–118.5)	2655	5907	449.5 (436.8–462.2)
Sensitivity analysis^e^						
2015	354	2889	122.5 (111.1−135.0)	1533	3690	415.4 (399.6−431.4)
2016	363	2972	122.1 (110.8−134.4)	1545	3777	409.1 (393.5−424.8)
2017	353	2994	117.9 (106.8−130.0)	1561	3785	412.4 (396.8−428.2)

^a^NDD-CKD patients at risk of anemia (year n, excluding patients with active cancer undergoing chemotherapy or patient with hematological cancers at December 31) − NDD patients with anemia of CKD (year n−1).

^b^Incidence estimated per year: number of incident NDD patients with anemia of CKD (year n)/number of NDD-CKD patients at risk of anemia (year n) − NDD patients with anemia of CKD (year n−1), per 1000 population.

^c^Prevalence estimated each year as: number of prevalent NDD patients with anemia of CKD/number of NDD-CKD patients at risk of anemia, per 1000 population.

^d^Main analysis: lookback period for the maximum time available between their index date and January 01, 2011.

^e^Sensitivity analysis: lookback period of 3 years prior to the calendar year of analysis. CKD, chronic kidney disease; CI, confidence interval; EGB, Echantillon Généraliste des Bénéficiaires; NDD, non-dialysis-dependent

When incidence and prevalence in the EGB population were used to approximate rates per year in the general French population, NDD-CKD and anemia of NDD-CKD incidence and prevalence were constant from 2015 to 2017 (**Tables 2 and 3**). In 2017, the incidence of NDD-CKD and anemia of NDD-CKD in the French population was 2.3 and 0.7 per 1000 population, respectively, and prevalence was 8.8 and 3.8 per 1000 population, respectively (**Tables 2 and 3**).

**Table 2:** Incidence and prevalence of NDD-CKD among the general population in France per year (2015−2017).

Year	Number of incident NDD-CKD patients	Number of EGB patients at risk of NDD-CKD^a^	Incidence^b^ estimate, per 1000 population (95% CI)	Number of prevalent NDD-CKD patients	Number of EGB patients	Prevalence^c^ estimate, per 1000 population (95% CI)
Main analysis^d^						
2015	1563	671,258	2.3 (2.2–2.4)	4741	675,349	7.0 (6.8–7.2)
2016	1603	686,384	2.3 (2.2–2.5)	5525	691,187	8.0 (7.8–8.2)
2017	1583	690,298	2.3 (2.2–2.4)	6126	695,706	8.8 (8.6–9.0)
Sensitivity analysis^e^						
2015	1563	671,258	2.3 (2.2−2.4)	3840	675,349	5.7 (5.5−5.9)
2016	1603	686,384	2.3 (2.2−2.5)	3962	691,187	5.7 (5.6−5.9)
2017	1583	690,298	2.3 (2.2−2.4)	3971	695,706	5.7 (5.5−5.9)

^a^EGB patients at risk NDD-CKD–NDD-CKD patients (year n−1).

^b^Incidence estimated per year: number of incident NDD-CKD patients (year n)/number of EGB patients at risk of anemia (year n)–NDD-CKD patients (year n−1), per 1000 population.

^c^Prevalence estimated each year as: number of prevalent NDD-CKD patients/number of EGB patients, per 1000 population.

^d^Main analysis: lookback period for the maximum time available between their index date and January 01, 2011.

^e^Sensitivity analysis: lookback period of 3 years prior to the calendar year of analysis. CKD, chronic kidney disease; CI, confidence interval; EGB, Echantillon Généraliste des Bénéficiaires; NDD, non-dialysis-dependent

**Table 3:** Incidence and prevalence of anemia of NDD-CKD among the general population in France per year (2015−2017).

Year	Number of Incident NDD patients with anemia of CKD	Number of EGB patients at risk of anemia^a^	Incidence^b^ estimate, per 1000 population (95% CI)	Number of prevalent NDD patients with anemia of CKD	Number of EGB patients at risk of anemia	Prevalence^c^ estimate, per 1000 population (95% CI)
Main analysis^d^						
2015	386	670,916	0.6 (0.5–0.6)	1992	672,193	3.0 (2.8–3.1)
2016	421	686,303	0.6 (0.6–0.7)	2328	687,878	3.4 (3.2–3.5)
2017	452	690,433	0.7 (0.6–0.7)	2655	692,252	3.8 (3.7–4.0)
Sensitivity analysis^e^						
2015	354	670,916	0.5 (0.5−0.6)	1533	672,193	2.3 (2.2−2.4)
2016	363	686,303	0.5 (0.5−0.6)	1545	687,878	2.2 (2.1−2.4)
2017	353	690,433	0.5 (0.5−0.6)	1561	692,252	2.3 (2.1−2.4)

^a^EGB patients at risk of anemia (year n, excluding patients with active cancer undergoing chemotherapy or patient with hematological cancers at December 31) − NDD patients with anemia of CKD (year n−1).

^b^Incidence estimated per year: number of incident NDD patients with anemia of CKD (year n)/number of EGB patients at risk of anemia (year n) − NDD patients with anemia of CKD (year n−1), per 1000 population.

^c^Prevalence estimated each year as: number of prevalent NDD patients with anemia of CKD /number of EGB patients at risk of anemia, per 1000 population.

^d^Main analysis: lookback period for the maximum time available between their index date and January 01, 2011.

^e^Sensitivity analysis: lookback period of 3 years prior to the calendar year of analysis. CKD, chronic kidney disease; CI, confidence interval; EGB, Echantillon Généraliste des Bénéficiaires; NDD, non-dialysis-dependent

Assuming an adult (age ≥18 years) French population of 53,466,197 (2020 census [26]), and using an NDD-CKD prevalence rate of 8.8 per 1000 population in 2017 from the present study, we estimated 470,503 patients with a diagnosis of NDD-CKD in France. Similarly, using the 2017 prevalence estimate of NDD-CKD-related anemia of 3.8 per 1000 population (**Table 3**, main analysis; **Table 4**), 203,172 patients are estimated to have anemia of NDD-CKD.

**Table 4:** Population estimates among patients with confirmed + possible NDD-CKD (main analysis^a^).

	Estimated number of patients
**Overall population** ^**b**^	**53,466,197**
Patients with NDD-CKD^c^	
Possible + Confirmed	2,256,274
Confirmed	470,503
Undiagnosed	1,785,771
Patients with NDD-CKD anemia	
Possible + Confirmed	684,367
Confirmed	203,172
Undiagnosed	481,195
Patients with stage 3‒5 NDD-CKD anemia^c^	
Possible + Confirmed	473,514
Confirmed	140,574
Undiagnosed	332,939

^a^Main analysis: lookback period for the maximum time available between their index date and January 01, 2011.

^b^Based on the projected general adult population in France in 2020 [26].

^c^Calculated based on the 69.2% of patients with stage 3–5 NDD-CKD anemia in this study; approximately only 80% of patients had CKD staging information available, as such these values will be underestimates. CKD, chronic kidney disease; CI, confidence interval; NDD, non-dialysis-dependent

Alternatively, in the sensitivity analysis, using the prevalence rate of NDD-CKD in 2017 for patients with markers of NDD-CKD over a three-year lookback period (5.7 per 1000 population; **Table 2**), there are an estimated 304,757 patients with a diagnosis of NDD-CKD in France. Similarly, using the 2017 prevalence estimate of anemia of NDD-CKD of 2.3 per 1000 population (**Table 3**), 122,972 patients are estimated to have anemia of NDD-CKD.

### Demographic and clinical characteristics

The age of NDD patients with anemia of CKD was stable during 2015−2017, with a median age of 83 years (**Table 5**). Approximately one-fifth of the patients were registered with long-term CKD (**Table 5**). Most patients with anemia of NDD-CKD had CKD stage 3, accounting for approximately one-third of incident and prevalent patients for each year, with increased proportions in 2016‒2017 (**Fig 3**). The proportion of incident and prevalent NDD patients with anemia of CKD and diabetes or cardiovascular disease was also relatively constant over time, with approximately one-third having diabetes and two-thirds having cardiovascular disease (**Table 5**).

**Table 5:** Demographic and clinical characteristics of patients with anemia of NDD-CKD.

		Incident NDD patients with anemia of CKD			Prevalent NDD patients with anemia of CKD		
Year		2015	2016	2017	2015	2016	2017
		N = 386	N = 421	N = 452	N = 1992	N = 2328	N = 2655
Age (years)	Mean (SD)	80.1 (12.2)	80.2 (11.9)	79.9 (12.2)	79.6 (12.9)	80.3 (12.5)	80.4 (12.4)
	Median	83	83	83	83	83	83
	min−max	26−104	27−101	28−101	19 −105	22−105	23−106
	Q1, Q3	75, 88	74, 88	74, 89	74, 88.5	74, 89	74, 89
Sex, n (%)	Male	204 (52.9)	221 (52.5)	2426 (54.4)	922 (46.2)	1060 (45.5)	1207 (45.5)
CMUc coverage, n (%)	Yes	8 (2.1)	17 (4.0)	18 (4.0)	63 (3.2)	85 (3.7)	108 (4.1)
CKD status^a^, n (%)	Yes	73 (18.9)	87 (20.7)	87 (19.3)	424 (21.3)	483 (20.8)	535 (20.2)
Diabetes, n (%)	Yes	130 (33.7)	129 (30.6)	143 (31.6)	728 (36.6)	848 (36.4)	968 (36.5)
CVD, n (%)	Yes	245 (63.5)	237 (56.3)	283 (62.6)	1321 (66.3)	1529 (65.7)	1736 (65.4)

^a^CKD long-term disease registration (Affection de Longue Durée). CKD, chronic kidney disease; CMUc, Couverture Médicale Universelle complémentaire (complementary universal health coverage); CVD, cardiovascular disease; NDD, non-dialysis-dependent

### Therapeutic management

Treatment of anemia in incident patients was analyzed for the first year of follow-up in patients with at least one treatment of interest (**Table 6**). Of patients with anemia of NDD-CKD, 42.5%–51.3% and 13.3%‒15.0% of patients received oral iron and ESA therapy, respectively (**Table 6**).

**Table 6:** Therapeutic management of incident patients with anemia of NDD-CKD during the first year after the index date (patients with ≥1 treatment of interest).

Treatment		2015	2016	2017
		N = 386	N = 421	N = 452
Iron^a^, n (%)	Oral	198 (51.3)	179 (42.5)	194 (42.9)
ESA^b^, n (%)	Erythropoietin	9 (2.3)	10 (2.4)	9 (2.0)
	Darbepoetin alfa	35 (9.1)	40 (9.5)	39 (8.6)
	Methoxypolyethylene glycol-epoetin beta	17 (4.4)	15 (3.6)	15 (3.3)
	None of the 3 treatments	330 (85.5)	358 (85.0)	392 (86.7)

^a^Intravenous iron use was not captured in the database from 2014 onwards.

^b^Excludes ESAs administered during any hospital stay. ESA, erythropoiesis-stimulating agent

### Disease progression

**Fig 4:**
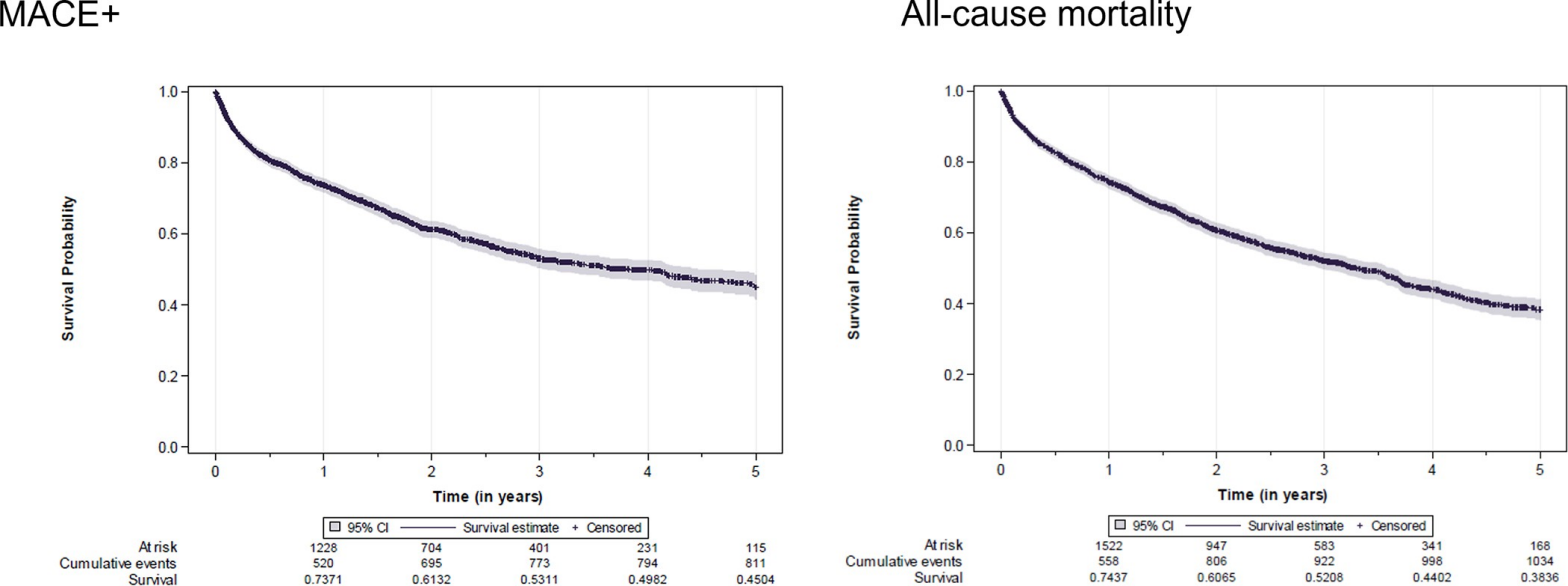
MACE+ and all-cause mortality Kaplan–Meier survival curves for incident NDD patients with anemia of CKD. MACE+, major adverse cardiovascular event plus (nonfatal stroke, nonfatal myocardial infarction, cardiovascular death, hospital admission for heart failure, or hospital admission for unstable angina).

The Kaplan–Meier survival curves for mortality and MACE+ are presented in **Fig 4**. Death from any cause was common. Median time to death was 3.3 years, and estimated survival rate was 38% at 5 years. The estimated median time to MACE+ was 3.9 years, and the event-free rate was approximately 45% over 5 years.

Occurrence of kidney transplantation prior to dialysis (dialysis being the end of follow-up) was infrequent: in the 5-year follow-up period, 13 patients received a kidney transplant, corresponding to an event-free rate of 99.0% in this study. Occurrence of dialysis affected a larger number of patients: 242 patients received dialysis during the 5-year follow-up, corresponding to an event-free rate of 83.1%.

### Exploratory outcome results

From the potential CKD analysis population, the machine learning algorithm detected 26,064 patients with “possible” NDD-CKD over the inclusion period (**Fig 2B**). This gave a total of 35,929 patients with confirmed (n = 9865) and possible (n = 26,064) NDD-CKD. When the definition of anemia was applied to this combined population, the number of patients with confirmed or possible NDD-CKD with anemia was 13,654, approximately three-fold more than the number identified using inpatient diagnostic codes and ALD registrations alone (n = 4848). Incidence and prevalence rates of anemia in the combined “possible” and “confirmed” CKD population were lower than the rates in the “confirmed” population alone (**Table 7**).

**Table 7:** Incidence and prevalence of anemia among patients with confirmed + possible NDD-CKD in the EGB database per year (2015–2017) (main analysis)^a^.

Year	Number of incident NDD patients with anemia of CKD	Number of NDD-CKD patients at risk of anemia^b^	Incidence^c^ estimate, per 1000 population (95% CI)	Number of prevalent NDD patients with anemia of CKD	Number of NDD-CKD patients at risk of anemia	Prevalence^d^ estimate, per 1000 population (95% CI)
2015	1735	18,423	94.2 (90.0−96.1)	6136	22,203	276.4 (270.5−282.3)
2016	1954	21,213	92.1 (88.3–96.1)	7627	26,193	291.2 (285.7−296.7)
2017	1916	21,886	87.5 (83.9−91.4)	8872	28,053	316.3 (310.8−321.7)

^a^Main analysis: lookback period for the maximum time available between their index date and January 01, 2011.

^b^NDD-CKD patients at risk of anemia: NDD-CKD patients at risk of anemia (year n, excluding patients with active cancer undergoing chemotherapy or patient with hematologic cancers at December 31) − NDD patients with anemia of CKD (year n−1).

^c^Incidence estimated per year: number of incident NDD patients with anemia of CKD (year n)/number of NDD-CKD patients at risk of anemia (year n) − NDD patients with anemia of CKD (year n−1), per 1000 population.

^d^Prevalence estimated each year as: number of prevalent NDD patients with anemia of CKD/number of NDD-CKD patients at risk of anemia, per 1000 population. CKD, chronic kidney disease; CI, confidence interval; NDD, non-dialysis-dependent

The estimated prevalence rate for confirmed + possible NDD-CKD as a proportion of the general population was 42.2 per 1000 population from 2017 (**S1 Table in S1 File**). Projecting this rate to an adult French population of 53,466,197 in 2020 [26], the estimated number of patients with NDD-CKD in France was 2,256,274, almost five-fold greater than the number identified by inpatient diagnostic codes and ALD registrations alone, and 1,785,771 more than our prior estimate of 470,503 based on the confirmed population (**Table 4**).

Based on the percentage of patients with stage 3–5 NDD-CKD in this study (69.2%), the proportion of the French population estimated to have anemia of “possible + confirmed” NDD-CKD stages 3–5 and the number of patients with undiagnosed NDD-CKD related anemia were 473,514 and 332,939, respectively (**Table 4**). However, given that only approximately 80% of patients in this study had staging information available, these proportions will be underestimates.

## Discussion

This retrospective database study is the first to provide reliable estimates of incidence and prevalence of NDD-CKD and associated anemia in France. Nearly 10,000 adult patients with confirmed NDD-CKD were identified in the EGB database, around half of whom had evidence of anemia, as determined by codification of prescribed treatment for anemia or hospitalization with an anemia diagnosis, rather than a laboratory diagnosis. Despite differing definitions of anemia, this proportion of patients with anemia of CKD is consistent with published studies from other countries reporting anemia in approximately 40–60% of pre-dialysis CKD patients [10, 11, 27–30].

The estimated incidence and prevalence of NDD-CKD anemia among NDD-CKD patients from the database remained relatively stable between 2015 and 2017. The annual incidence over this period was 108.7−114.7 per 1000 population, similar to the 2016 incidence (114 per 1000 population) reported for Italian patients with stage 3−5 NDD-CKD [31]. Since the EGB is representative of the French population (in terms of age, sex, and healthcare consumption), the number of NDD-CKD patients in the EGB population was used to estimate NDD-CKD prevalence nationally. We estimate that there are almost 475,000 patients with a diagnosis of NDD-CKD in France.

The unpredictable rate of CKD progression and the silent nature of early CKD stages makes early detection a challenge [32]. Data from the Centers for Disease Control assessing undiagnosed CKD in the United States has shown that, even among adults at high risk (≥15%) of kidney failure within 5 years, only 50% were aware of having CKD [33]. Given that patients in this study with confirmed NDD-CKD were identified using hospital diagnosis or ALD codes, we expected that less severe stages of the disease (particularly stages 1–2) would be underestimated. Indeed, the Global Burden of Disease estimated that the global prevalence of CKD in 2017 was 9.1% (including approximately 0.05% for CKD patients with dialysis or kidney transplantation); of which, over half belonged to patients with CKD stages 1–2 [3]. By applying a machine learning tool, we estimated that there may be close to 1.8 million patients in France with undiagnosed NDD-CKD (or otherwise not identified and captured in the EGB database) and, among these individuals, 332,939 may have associated anemia. While the total estimated number of diagnosed plus undiagnosed individuals with NDD-CKD (around 2.26 million) in our study is less than that estimated by the Global Burden of Disease analysis for 2017 in France (~6 million people) [3], it aligns closely with the 2.45 million estimated by a previous study (MONA LISA), which used prevalence data from cross-sectional surveys in adults aged 35–74.9 years and standardized to the French population [34].

Characterization of patients with confirmed NDD-CKD anemia revealed a median age of 83 years, which is older than that previously reported for dialysis-dependent CKD patients in France [35, 36]. Since anemia is known to increase substantially with age [37], the older age profile of the NDD-CKD anemia population retrieved from the EGB database suggests the reliability of the data. However, as the study population included hospitalized patients with NDD-CKD, the age profile of these data could also reflect the fact that older patients are hospitalized more frequently than younger patients, and hence anemia was identified in a greater proportion of older patients. Analysis of clinical characteristics found that 65−66% of prevalent NDD patients with anemia of CKD also had cardiovascular disease. This is consistent with a real-world study conducted across Europe (France, Germany, Italy, Spain, and UK) in which approximately two-thirds of patients with anemia had concomitant CV conditions [11]. The same study also revealed that cardiovascular conditions in patients with anemia of CKD were significantly associated with reduced quality of life and work productivity, whereas no such associations were evident in non-anemic patients or the total CKD population [11].

Treatment options for anemia of CKD include oral iron replacement and ESAs. Among patients with NDD-CKD-related anemia, less than half received oral iron and approximately only 15% received ESAs. This is further supported by the Chronic Kidney Disease Outcomes and Practice Patterns (CKDopps) study, a prospective cohort study of patients with NDD-CKD-related anemia from 2013 to 2018: among French patients with Hb <10 g/dL, 34% were not prescribed ESA or iron therapy in the 3 months following Hb measurement [15]. A retrospective cohort study evaluating the prevalence and incidence of stage 3a–5 NDD-CKD-related anemia (defined as Hb <13 g/dL in males and <12 g/dL in females) in Italy from 2014 to 2016 found that ≤15.5% of eligible patients received ESA treatment [38].

While this study does not capture inpatient administration of either intravenous iron or ESAs, our results are consistent with other real-world studies suggesting that anemia treatment is initiated in a limited number of patients with NDD-CKD-related anemia, especially those with stage 3 CKD [31, 39]. The use of real-world data routinely collected from a large, nationally representative database [17] to provide real-world evidence for the incidence and prevalence of NDD-CKD anemia in France is a strength of our study. Furthermore, this study is the first of its kind in France to generate results indicating that a substantial proportion of French NDD-CKD patients is underdiagnosed and thus managed very late in their disease.

The database also had several limitations. The EGB database did not include clinical laboratory/imaging data or outpatient medical diagnoses (e.g., consultations or examinations); as such, the definitions used for CKD (by diagnostic codes and/or disease registration) and anemia (by treatment and/or hospital admission for anemia) could not use available laboratory data. Treatments for anemia or anemia recorded during hospitalization may not have been caused by NDD-CKD (although we can confirm that chemotherapy was not a potential cause, as patients with a record of chemotherapy or recent hematological cancer were excluded from this study) and hence may have inflated the prevalence rates of NDD-CKD-related anemia reported herein. Furthermore, the EGB database did not capture diagnostic codes for outpatients, which may have meant that some patients predicted to have undiagnosed NDD-CKD by our machine learning model were already diagnosed.

The database also captures all dispensed and reimbursed healthcare consumption; however, although not an objective of the current study, prescribed but non-reimbursed treatments are not included in the database, which precludes assessment of whether exposure to certain treatments fluctuated according to their compliance. Drugs included in the Diagnosis-Related Group French hospital payment system [40] (based on the Groupe Homogène de Malades) were not identifiable and therefore were not available for analysis; this applied to intravenous iron therapy from 2014 onwards as well as any ESAs administered during a hospital stay, although subcutaneous ESA treatment administered in an outpatient setting was still captured. Additionally, over-the-counter drugs were not captured in the EGB database used in this study. As medications used in patients on dialysis are not captured in EGB, the target population included in this study was restricted to patients with NDD-CKD only. Lastly, estimates for overall CKD prevalence in France were derived from the GBD study in 2017 [3], as more recent data are not yet available.

As a retrospective observational study that used pseudo-anonymized data derived from administrative claims database, bias may exist regarding diagnoses recorded in the database for long-term diseases and for hospital diagnoses. However, this should be minimized since long-term disease diagnoses were approved by the NHI, and diagnosis codes were chosen following national guidelines. Additionally, the inclusion of only patients having undergone a hospitalization involving CKD or who were on the long-term disease list could lead to enrichment of more severe or co-morbid patients and an underestimation of the confirmed NDD-CKD population.

The NDD-CKD population may have been underestimated due to a limited lookback period. Patients with CKD stages 1 and 2 may also be less likely to be hospitalized and/or codified, and therefore be more likely to be under-represented, with the prevalence of patients in stages 3–5 being more reliable. This is consistent with the lower number of confirmed NDD-CKD patients reported in our study versus our pre-study estimate, (although our estimate included both NDD and DD-CKD patients, **S2 Table in S1 File**). Finally, subclassification of CKD stage 3 patients into 3a and 3b was not reported in the database. The CKD stage was also only captured for approximately 4 out of 5 patients.

## Conclusions

This retrospective analysis provides the first estimation based on patient-level data of the incidence and prevalence of NDD-CKD anemia in France, as well as an indication of the number of patients with potentially undiagnosed CKD. Initiatives to better identify and treat NDD-CKD anemia in the French population may improve outcomes, particularly for the two-thirds of patients with co-existing cardiovascular disease.

## Supporting information

S1 File (PDF)
