# Supplementary material for: Incidence, prevalence, and treatment of anemia of non-dialysis-dependent chronic kidney disease: A retrospective database study in France
Source: PLoS One. 2023 Jul 5;18(7):e0287859. doi: 10.1371/journal.pone.0287859 (PMC10321647; doi:10.1371/journal.pone.0287859)
Supplement: S1 File — (PDF) [file pone.0287859.s001.pdf]

SUPPORTING INFORMATION

Supplementary methods

Primary objective

To estimate the incidence and prevalence of NDD CKD anemia among NDD CKD patients for each year during the observation period (2012–2017), the number of NDD CKD patients at risk of anemia per year of the observation period (2012–2017) was computed. This was defined as the annual number of NDD CKD patients excluding patients with active cancer undergoing chemotherapy or patients with hematologic cancers on December 31 of the current year.

An incident NDD CKD anemia patient was defined as a patient with a first marker of anemia during the follow-up period (i.e., 2012–2017), and without prior marker of anemia since 2011 (the date when data is available for the purpose of this study). Incidence was estimated each year as: number of new NDD CKD anemia patients in the year n / number of NDD CKD patients at risk of anemia in the year n (NDD CKD patients – NDD CKD patients with active cancer undergoing chemotherapy or patient with hematologic cancers in the year n − NDD CKD anemia patients in the year n−1).

Prevalence was estimated each year as: number of NDD CKD anemia patients in the year n / (number of NDD CKD patients in the year n − patients with active cancer undergoing chemotherapy or patient with hematologic cancers in the year n). Once a patient was identified as anemic in a given year of the follow-up period (i.e., 2012–2017), the patient was considered anemic for the rest of the follow-up period.

Secondary objectives

Incidence and prevalence of NDD CKD anemic patients for each year during observation period (2012–2017) among the general population in France was assessed through the comparison of the following counts: the number of incident/prevalent NDD CKD anemic patients per year of the observation period (2012-2017) as computed previously, and the number of persons in France per year of the observation period (2012–2017), defined as the annual number of patients among EGB database excluding patients with active cancer undergoing chemotherapy or patients with hematologic cancers at index date.

Incidence was estimated each year as: number of new NDD CKD anemic patients in the year n / number of EGB patients at risk of anemia in the year n (EGB patients - patients with active cancer undergoing chemotherapy or patients with hematologic cancers in the year n − NDD CKD anemic patients in the year n−1). Prevalence was estimated each year as: number of NDD CKD anemic patients in the year n / (number of EGB patients in the year n − patients with active cancer undergoing chemotherapy or patients with hematologic cancers in the year n).

Study size

To ensure the EGB database would provide a sufficient sample size for our study, we estimated the number of cases of CKD patients captured in the database using unadjusted numbers from a previous review of CKD prevalence across the European general population [1]. Estimates of prevalence were provided by patient age group; however, estimates from the Netherlands were used as a substitute for the 20–44 years age group, as no estimates were available from the French studies for this age bracket and similar findings to France were observed for all other age groups. From this analysis, we estimated that the EGB database would have 53,991 adult cases of CKD (DD and NDD) (**Table S2**).

Similarly, we looked at the distribution of CKD reported by stage in an Italian study [2], combined with US estimates for anemia [3]. From this, we estimated that of the predicted 53,991 adult cases of CKD, 7703 would have a record of anemia (**Table S3**). These crude estimates of CKD anemia cases in the EGB should be considered in terms of order of magnitude and not exact figures; however, we expect the EGB database to provide a sufficient number of cases for estimating incidence and prevalence with good precision, as well as enabling accurate summaries of the characteristics of the cohort.

References

1. Brück K, Stel VS, Gambaro G, Hallan S, Völzke H, Ärnlöv J, et al. CKD prevalence varies across the European general population. J Am Soc Nephrol American Society of Nephrology; 2016; 27: 2135–47.

2. De Nicola L, Zoccali C. Chronic kidney disease prevalence in the general population: heterogeneity and concerns. Nephrol Dial Transplant 2016; 31: 331–5.

3. Stauffer ME, Fan T. Prevalence of anemia in chronic kidney disease in the United States. PLoS One 2014; 9: e84943.

Machine learning algorithm – step-by-step process

Step 1 – Input

Detection of the potential CKD patient population was conducted based on the following assumptions:

- The potential CKD population contains CKD patients.
- We take into account all the relevant variables related to CKD.
- Most of the potential population is not CKD.

Potential CKD patients were extracted, based on the following variables:

- Sex (index date)
- Anemia
- Number of hospitalizations for renal diseases
- Duration of hospitalizations for renal diseases
- Number of general practitioner visits
- Treatments: furosemide (Lasix/Lasilix® >80mg), polystyrene sulfonate (Resikali®, Kayexalate®), sevelamer (Renvela®, Renagel®), lanthanum (Fosrenol®), sucroferric oxyhydroxide (Velphoro®), calcium acetate (Phosphosorb®), calcium bicarbonate, alfacalcidol (Unalfa®), calcitriol (Rocaltrol®). Treatments were grouped into ATC classes
- Biological exams: creatinine, proteinuria, calcium, phosphate, parathyroid hormone concentration, renal biopsy and/or renal echography procedures

After discussions with the scientific committee, the following variables were removed for the training of the algorithm:

- Age (index date): Removed because of the bias that it could replicate
- CMUc (index date): Removed because this is not relevant for this analysis

- CCI (Charlson Comorbidity Index): Removed because of the bias that it could replicate
- Comorbidities: Removed because of the bias that it could replicate
- Anemia: Removed because it should be studied after the training of the algorithm
- Hospitalizations for reasons other than renal failure: Removed because of the bias that it could replicate
- Outpatient visits to the hospital, whatever the reason: Removed because of the bias that it could replicate
- Albumin: Removed because of the bias that it could replicate

Variables creating a bias are, however, studied in the last step to look at the similarity between the groups.

Step 2 - Algorithm training

The algorithm is trained to find the outliers (i.e., possible CKD patients) in the potential population. These outliers are identified using a One-Class-SVM, an unsupervised algorithm that learns a decision function for novelty detection. It learns how to construct a spherical boundary in a feature space around the data. The volume of this hypersphere is minimized to minimize the effect of incorporating outliers in the solution. Thus, the algorithm learns automatically how to differentiate normal patients and outlier patients. This was implemented using the scikit-learn Python package (0.23.2 revision).

Step 3 – Validation of the Possible CKD population

The algorithm compared “patient profiles” (decision functions based on the variables in the model) with patients in the confirmed CKD population in order to see if detected CKD patients (possible CKD) are similar to the confirmed CKD patients.

For the validation of the algorithm, two main points were considered:

- Most of the potential population is assumed not to be CKD, so the proportion of patients classified as CKD by the algorithm in the potential CKD population should be low.
- The proportion of confirmed CKD patients classified as CKD by the algorithm should be higher than the proportion of possible CKD among potential CKD, because patients in this population are actually CKD.

The validation step allows us to have the following information:

- 26,064 outlier patients (i.e., possible CKD patients) were detected in the potential CKD population. This number is low (21%), which means that the algorithm is selective in this population and only finds atypical patients.
- When we use the same algorithm on the confirmed CKD population, 4,044 patients were classified as having CKD. This number is high (65%), which means that the patients found in the potential population correspond to most of the patients of the confirmed CKD population.

With these two metrics, we can say that the algorithm selects relevant patients.

Description of possible CKD Patients

Following assessment of the possible CKD population found by the algorithm, no bias in the possible CKD population was observed regarding patient age or comorbidity status. Furthermore, the populations of confirmed CKD and the possible patients with CKD were similar; we therefore conclude that our algorithm is relevant and allowed us to identify patients with CKD.

S1 Table: Incidence and prevalence of confirmed and possible NDD CKD among the general population in France per year

Year	Number of Incident NDD patients with anemia of CKD	Number of EGB patients at risk of anemia[a]	Incidence[b] estimate, per 1000 population (95% CI)	Number of prevalent NDD patients with anemia of CKD	Number of EGB patients at risk of anemia	Prevalence[c] estimate, per 1000 population (95% CI)
Main analysis[d]						
2015	5,708	668,485	8.5 (8.3–8.8)	23,188	675,349	34.3 (33.9–34.8)
2016	5,730	682,636	8.4 (8.2–8.6)	27,340	691,187	39.6 (39.1–40.0)
2017	3,924	685,560	5.7 (5.5–5.9)	29,343	695,706	42.2 (41.7–42.7)
Sensitivity analysis[e]						
2015	5,708	668,485	8.5 (8.3–8.8)	15,403	675,349	22.8 (22.5–23.2)
2016	5,730	682,636	8.4 (8.2–8.6)	15,895	691,187	23.0 (22.6–23.4)
2017	3,924	685,560	5.7 (5.5–5.9)	14,466	695,706	20.8 (20.5–21.1)

[a]EGB patients at risk NDD-CKD – NDD-CKD patients (year n−1). [b]Incidence estimated per year: number of incident NDD-CKD patients (year n)/number of EGB patients at risk of anemia (year n) – NDD-CKD patients (year n−1), per 1000 population. [c]Prevalence estimated each year as: number of prevalent NDD-CKD patients/number of EGB patients, per 1000 population. [d]Main analysis: lookback period for the maximum time available between their index date and January 01, 2011. [e]Sensitivity analysis: lookback period of 3 years prior to the calendar year of analysis. CKD, chronic kidney disease; CI, confidence interval; EGB, Echantillon Généraliste des Bénéficiaires; NDD, non-dialysis-dependent

S2 Table: Estimated number of CKD cases in the EGB database

Country	Age group (years)	Prevalence (%)	95% CI	Population distribution in France (%)	EGB estimated sample population (n=780,000)	EGB estimated number of CKD cases (prevalence x sample)
Netherlands	20-44	4.5	3.2-5.8	29.8	232,440	10,460
France	45-64	3.9	3.6-4.1	26.1	203,580	7,940
France	65-74	15.9	12.3-19.5	10.8	84,240	13,394
France	75-84+	30.6	27.3-33.9	9.3	72,540	22,197
Total	-	-	-	-	592,800	53,991

CKD, chronic kidney disease; EGB, Echantillon Généraliste des Bénéficiaires

S3 Table. Estimated number of anemia CKD cases in the EGB database

CKD stage	Proportion assumed among CKD 1-5 (%)	EGB estimated CKD population	Proportion assumed with anemia (%)	EGB estimated CKD anemia population
1	37.4	20,218	8.4	1,698
2	21.6	11,641	12.2	1,420
3	37.0	19,988	17.4	3,478
4	2.3	1,225	50.3	616
5	1.7	919	53.4	491
Total	-	53,991	-	7,703

CKD, chronic kidney disease; EGB, Echantillon Généraliste des Bénéficiaires
